# Hsa_circ_0000437 promotes the progression of rheumatic valvular heart disease by activating the mitogen-activated protein kinase signaling pathways after sponging let-7f-5p and targeting RAS-like proto-oncogene B

**DOI:** 10.1007/s13577-025-01331-7

**Published:** 2025-12-15

**Authors:** Linwen Zhu, Ni Li, Huoshun Shi, Jie Song, Zhongjie Fang, Qinbo Shen, Xiuying Zhu, Yanggang Dan, Guofeng Shao, Lebo Sun

**Affiliations:** 1 Department of Cardiovascular Surgery, The Affiliated Lihuili Hospital of Ningbo University, Ningbo City, 315041 Zhejiang China; 2 Institute of Pharmaceutics, College of Pharmaceutical Sciences, Zhejiang University, Hangzhou, China; 3 Department of Thoracic Surgery, The Affiliated Lihuili Hospital of Ningbo University, Ningbo City, 315041 Zhejiang China

**Keywords:** Hsa_circ_0000437, Let-7f-5p, RAS-like proto-oncogene B (RALB), Mitogen-activated protein kinase (MAPK) signaling pathways, Pathogenesis, Rheumatic valvular heart disease (RVHD)

## Abstract

Circular RNAs (circRNAs) are involved in cardiovascular disease development and progression. We identified has_circ_0000437 from three pairs of clinical samples using a circRNA microarray and predicted the has_circ_0000437 sponge let-7f-5p using bioinformatics. We expanded the sample to confirm the expression, correlation, and diagnostic value of has_circ_0000437, let-7f-5p and RAS-like proto-oncogene B (RALB) in rheumatic valvular heart disease (RVHD). The effects of has_circ_0000437 and let-7f-5p on biological function were observed in hVICs cells. To explore the molecular pathogenesis of has_circ_0000437 in the RVHD process, the target binding protein RAS-like proto-oncogene B (RALB) of has_circ_0000437 was obtained using label-free mass spectrometry and parallel reaction monitoring. The influence of the has_circ_0000437/let-7f-5p/RALB axis on the expression of mitogen-activated protein kinase (MAPK)-related proteins in the inflammatory signaling pathway predicted by Kyoto Encyclopedia of Genes and Genomes (KEGG) during RVHD was measured using Western blotting. We found that let-7f-5p was at low expression and negatively correlated with has_circ_0000437, which had a diagnostic value with an area under the curve of 0.998 after the combined diagnosis. Has_circ_0000437 and let-7f-5p had binding sites, and let-7f-5p alleviated the effect of has_circ_0000437 on the proliferation, migration, and cycle progression of hVICs. The inhibitory effect of has_circ_0000437 on hVICs apoptosis was diminished. Mechanism studies showed that has_circ_0000437 promotes the MAPK pathway through has_circ_0000437/let-7f-5p/RALB axis. These findings suggest that the has_circ_0000437/let-7f-5p/RALB axis promotes the MAPK pathway, proliferation, migration, and cycle progression in RVHD, inhibiting the apoptosis process, thereby promoting RVHD development. This pathway may suggest a target for RVHD diagnosis and treatment.

**Supplementary Information:**

The online version contains supplementary material available at 10.1007/s13577-025-01331-7.

## Introduction

Rheumatic heart disease is associated with group A beta-hemolytic streptococcal infections and leads to immune and cellular responses in cardiac tissue, ultimately affecting heart valve structure and function [1, 2]. Heart valve involvement often results in the development of RVHD [3, 4]. The morbidity and mortality associated with RVHD decrease yearly worldwide; nevertheless, the disease continues to impose substantial burdens in developing countries, such as China and India [5]. The early clinical manifestations of RVHD are usually not evident and lack specific diagnostic markers [6]. Symptoms often accompany stenosis or incomplete valve closure, requiring medical treatment or surgery [7], and no precise therapeutic target has been found.

Circular RNA (circRNA) is a class of RNA molecules with a closed circular structure. They are found in various organisms and have higher stability than linear RNA [8, 9]. Their biological functions include the coexistence of miRNAs as competing endogenous RNAs (ceRNAs) after sponge adsorption, targeted binding of RNA-binding proteins, translation, and regulatory transcription [10, 11]. CircRNAs have specific biological functions in tumors [12–14], and they participate in cardiovascular diseases, such as myocardial hypertrophy, myocardial infarction, and heart failure [15–17]. CircRNAs have been used as biomarkers to diagnose cardiovascular diseases [18–20].

Nevertheless, there is little research on the relationship between circRNA and RVHD, and effective targets for RVHD diagnosis and treatment are needed. In previous studies, our research group performed circRNA microarray analysis on the plasma of RVHD patients and uploaded the results to the NCBI GEO database (GSE168932, https://www.ncbi.nlm.nih.gov/geo/query/acc.cgi?acc=GSE168932) [21]. We found that hsa_circ_0000437 can be used as a marker to diagnose RVHD. It also promotes RVHD by promoting the proliferation, migration, and cycle progression while inhibiting apoptosis of hVICs cells immortalized from the heart valve tissue of RVHD patients. In our previous study, we determined the diagnostic value of let-7f-5p absorbed by the hsa_circ_0000437 sponge in RVHD and its influence on the biological function in RVHD [21]. To explore how hsa_circ_0000437 mediates RVHD progression, we identified the targeted binding protein RAS-like proto-oncogene B (RALB) of hsa_circ_0000437 using mass spectrometry identification and parallel reaction monitoring (PRM). The mechanism of hsa_circ_0000437 promoting RVHD progression was revealed by examining the changes in expression levels of target protein RALB and the related proteins in the mitogen-activated protein kinase (MAPK) signaling pathway identified by GO and KEGG in circRNA microarray analysis. Our findings suggest a target for RVHD therapy.

## Materials and methods

### Clinical specimen collection and storage

We considered 42 patients with RVHD, 42 with non-rheumatic valvular disease (NRVD), and 42 healthy controls admitted to the Department of Cardiovascular Surgery, The Affiliated Lihuili Hospital of Ningbo University. The admission and exclusion criteria and the information about demographics for these patients were detailed previously [21]. The Ethics Committee of The Affiliated Lihuili Hospital of Ningbo University, approved the ethics-related study (number KY2021PJ191). This study has obtained the informed consent of each patient and healthy person.

After centrifugation, plasma was placed in RNAse-free Eppendorf tubes, and plasma samples were stored at –80 °C ultra-low before freeze-thawing.

### CircRNA microarray assays and bioinformatics analysis

As in the previous study, Arraystar Human circRNA microarray (Arraystar, USA) was used to analyze three pairs of clinical samples [21]. The microarray results were sorted and analyzed using R software, and volcano plots, hierarchical clustering heatmaps, scatter plots, and box plots were drawn to display the results of the microarray analysis.

Based on the microarray analysis, we predicted the sponge miRNAs and target proteins of hsa_circ_0000437. Using GO and KEGG analysis, we used DIANA–miRPath online software to reveal the relevant biological functions and molecular signaling pathways of hsa_circ_0000437 in RVHD progression.

### Identification of targeted binding proteins using label-free mass spectrometry

After lysis, protein quantities were measured using BCA, precipitated in acetone, redissolved, restore, alkylated, and enzymatically digested. The samples were prepared for mass spectrometry by removing SDC and desalting polypeptide. The nano-UPLC separation procedure was as follows: 2 μg peptides were separated from each group using the nano-UPLC liquid phase system EASY–nLC1200 and measured using a Q-Exactive mass spectrometer. LC–MS/MS mass spectrometry was used for quantitation, the relevant parameters are as follows, mass spectrometry analysis duration: 120 min/sample; positive ion detection mode, parent ion scanning range: 350–1600 m/z. MaxQuant analysis and LFQ quantification were conducted simultaneously. Statistical tests and GO/KEGG bioinformatics and STRING database analysis were performed. In addition, combined with the PRM results; we combined the RNA pull-down assay conducted to confirm the direct and binding regulatory relationship between hsa_circ_0000437 and RALB. The RNA Immunoprecipitation (RIP) experiment was also conducted to investigate whether RALB directly interacted with hsa_circ_0000437.

### PRM technique verifies targeted binding proteins

After separation using nano-UPLC, PRM was performed; after LC–MS/MS mass spectrometry and analysis, the PRM data were imported into Skyline for transition extraction, we have deposit data at the Panorama Public Repository (https://panoramaweb.org/Panorama%20Public/project-begin.view). The hsa_circ_0000437 target binding protein was found to be RALB.

### RNA pull-down assay

The biotin (Beyotime, China) was transcribed with synthetic hsa circ0000437 and let-7f-5p probe (Tsingke, China) to prepare biotin-labeled RNA probes. The biotin-labeled RNA probe was then incubated with cell lysate to bind the probe to the target RNA, and the biotin-labeled RALB negative control (RALB-NC) hsa_circ_0000437 antisense sequence and miRNA-negative control (miR-NC) were used as controls, respectively. Then, the biotin-labeled probe and its binding RNA complex were captured by streptavidin magnetic beads (Beyotime, China). Eluent (Beyotime, China) continued to eluate RNA–RNA complexes from magnetic beads, and qRT–PCR was used to analyze target RNAs and their interacting RNAs. The statistical method adopted was the independent sample* t* test.

### RIP assay

The RALB antibody (Proteintech, China) was incubated overnight with magnetic beads (Beyotime, China), then the cells were lysed with cell lysate (Beyotime, China). The lysate was then mixed with the antibody–magnetic beads and incubated for 3 h. The RNA adsorbed by the magnetic beads was extracted and IgG (Beyotime, China) was used as a control. Finally, the enrichment was detected by qRT–PCR. The statistical method adopted was the independent sample* t* test.

### qRT–PCR assay

Total RNA was extracted from plasma samples using TRIzol LS reagent (Invitrogen, USA). After performing the RNA quality test, total RNA was reverse-transcribed into cDNA using a reverse transcription kit (GenePharma, China). An amplification kit (Promega, USA) was used for qRT–PCR amplification to detect the expression of let-7f-5p and RALB. U6 and GAPDH were used as the external reference control of let-7f-5p and RALB, respectively. The let-7f-5p and RALB primer sequences were as described [22, 23]. Let-7f-5p and RALB expression were measured using the relative quantitative method and were calculated using the 2^−ΔCq^ method; higher 2^−ΔCq^ indicated significant expression, the statistical method adopted was the one-way ANOVA. The primer sequence is shown in Table S1.

### Cell culture and transfection

After immortalizing hVICs cells from heart valve mesenchymal cells in patients with RVHD, our research group’s previous studies described the cell culture [21], transfection methods are implemented based on relevant research[22, 23].

### Cell-counting kit 8 (CCK-8) assay

The transfected cells (5 × 10^3^) were inoculated in 96-well plates and cultured for 1, 2, 3, 4, and 5 days. We added 10 μL CCK-8 reagent (Dojindo, Japan) and incubated for 3 h. The optical density was measured and recorded using an enzyme labeling instrument. The statistical method adopted was the one-way ANOVA.

### Transwell assay

Transfected hVICs cells (8 × 10^4^ in 200 μL) were added to the upper chamber of transwell plates (Costar, USA), and 500 μL cell culture medium containing 20% fetal bovine serum (Gibco, USA) was added to the lower chamber. The cells were incubated for 24 h, fixed with 4% paraformaldehyde (Aladdin, China), and stained with 0.1% crystal violet (Sinopharm, China). The statistical method adopted was the one-way ANOVA.

### Apoptosis assay

After transfection, the cells were digested with EDTA-free trypsin (Gibco, USA) and re-suspended in a binding buffer (Multi Sciences, China). After standing at room temperature, they were dyed with an Annexin V-FITC/PI Apoptosis Kit (Multi Sciences, China) and shaded in the dark for 15 min. Finally, apoptosis was measured using FACSCalibur flow cytometry (Becton Dickinson Co., USA). All steps were performed following the manufacturer’s instructions. The statistical method adopted was the one-way ANOVA.

### Cell cycle assay

Before transfection, cells were starved and cultured in a serum-free medium to synchronize the cell cycle. The cells were collected and cleaned with phosphate-buffered saline, fixed with 70% ethanol, and maintained at –20 °C overnight. The cells were removed, rinsed with pre-cooled phosphate-buffered saline, and stained using 1 mL PI/RNase staining buffer (Multi Sciences, China). Cells were incubated in the dark for 30 min. Cell cycle distribution was measured using FACSCalibur flow cytometry (Becton Dickinson Co., USA). All steps were performed following the manufacturer’s instructions. The statistical method adopted was the one-way ANOVA.

### Dual‑luciferase reporter assay

After constructing wild-type or mutant hsa_circ_0000437 and RALB reporter plasmids (GenePharma, China) containing let-7f-5p binding sites, the reporter plasmid and let-7f-5p mimics were co-transfected into hVICs cells in 24-well plates according to the manufacturer’s instructions (GenePharma, China). The relative luciferase activity was measured. The statistical method adopted was the independent sample *t* test.

### Western blotting analysis

The transfected cells were cleaved with lysate solution (Solarbio, China), and the proteins were extracted and quantified with a Bradford detection kit (Beyotime, China). Protein samples were separated using 12% sodium dodecyl-polyacrylamide gel electrophoresis; then the membrane was transferred and incubated with primary antibody, washed in tris-buffered saline with Tween-20, incubated with secondary antibody, and rewashed in the same buffer. Protein signal intensity was measured using a Clinx GenoSens 1600 gel imaging analysis system (Clinx, China) after adding WesternBright ECL horseradish peroxidase (Advansta, USA) chemiluminescence solution. MAPK14 (Cat No. 14064–1-AP), extracellular regulated protein kinases (ERK) (Cat No. 11257–1-AP), c-Jun N-terminal kinase (JNK) (Cat No. 51153–1-AP), RAS-like proto-oncogene B (RALB) (Cat No. 12340–1-AP), and goat anti-rabbit IgG and goat anti-mouse IgG secondary antibody were purchased from a subsidiary of Proteintech Wuhan Sanying Biotechnology Co., LTD (China). Each experiment was repeated three times, the statistical methods adopted were independent sample *t* test or analysis of variance.

### Statistical analysis

All data were expressed as mean ± standard deviation and analyzed using Social Science Statistics Program 20.0 (IBM, USA) and GraphPad Prism 6.0 (GraphPad Software, USA). Appropriate statistical methods were used to calculate the differences between groups. Differences where *P* < 0.05 were considered statistically significant.

## Results

### circRNA expression profile and bioinformatics analysis in RVHD patient plasma

**Fig. 1:**
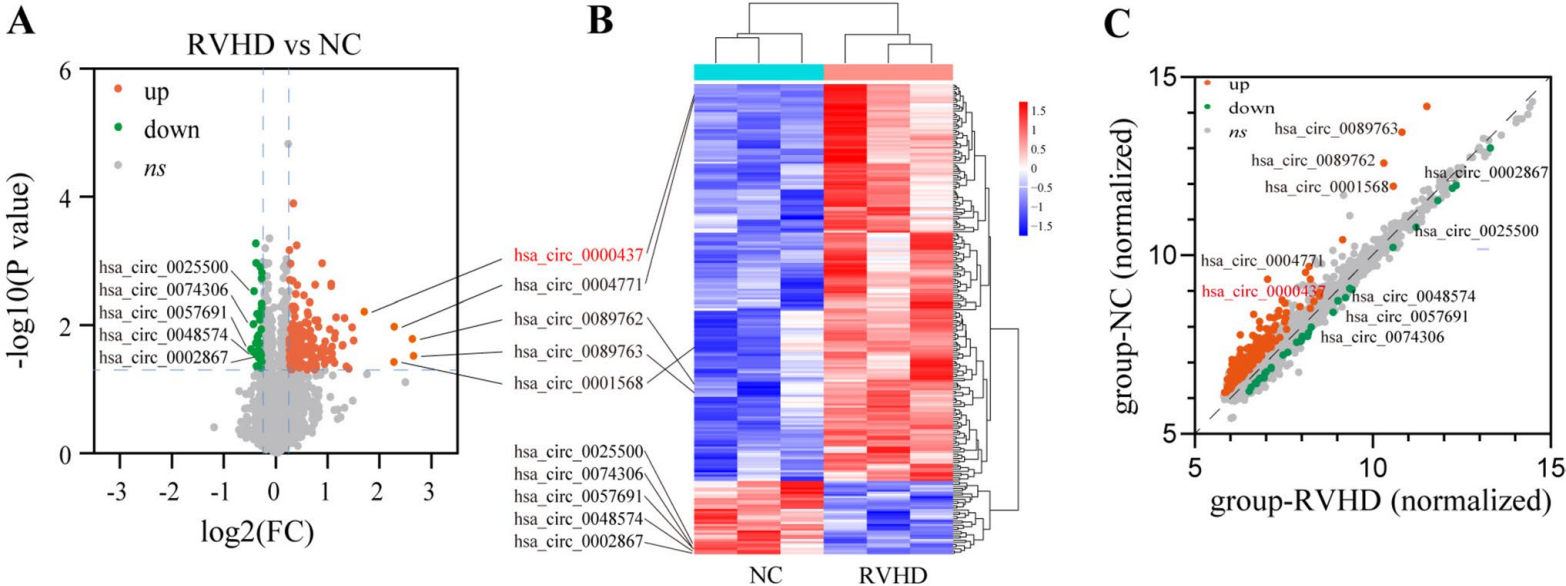
circRNA microarray data (*n* = 3). **A** Volcano plot of differential circRNA expression between RVHD patients and normal controls (the red dots represent the representative up-regulated circRNAs, and the green dots represent the representative down-regulated circRNAs, fold-change = 1.2, *P* < 0.05). **B** Hierarchical clustering heatmap of circRNAs expression differences between RVHD patients and normal controls, and the representative up-regulated and down-regulated circRNAs in RVHD patients and normal controls were marked. **C** Scatter plot of the difference in circRNA expression between RVHD patients and normal controls, and the representative up-regulated and down-regulated circRNAs in RVHD patients and normal controls also were marked. *n* stands for replicate samples

Differential expression profiles of circRNA in plasma between RVHD patients and controls were evaluated using high-throughput human circRNA microarrays. We detected 9295 circRNAs and uploaded them to NCBI’s GEO database (GSE168932, https://www.ncbi.nlm.nih.gov/geo/query/acc.cgi?acc=GSE168932). CircRNAs were differentially expressed in RVHD patients, and control plasma was characterized. Cluster analyzed using various statistical descriptions, including volcano map (fold-change = 1.2, *P* < 0.05)) (Fig. 1A), cluster map (Fig. 1B), and scatter plot (Fig. 1C). These characterizations, respectively, represent the expression level of circRNA, the expression pattern of circRNA, and the differentially expressed circRNA. We narrowed the range and identified 81 circRNAs with significant differences (fold-change = 1.5, *P* < 0.05). Table S2 shows 81 differentially expressed circRNAs. Based on our previous research findings, hsa_circ_0000437 can be used as a reliable biomarker for RVHD [21]. This study continues to investigate its therapeutic target in RVHD.

Based on the results of circRNA microarray analysis, we predicted the sponge miRNAs and target proteins of hsa_circ_0000437 using bioinformatics. We identified 62 sponge miRNAs that might bind to hsa_circ_0000437 (Fig. S1A). These include miR-502-5p, let-7a-5p, let-7b-5p, let-7c-5p, and let-7f-5p, which were previously predicted using Circbank and circular RNA interaction software [21]. We also identified 165 target proteins that may bind to hsa_circ_0000437 (Fig. S1A). These target proteins need binding verification; therefore, we considered them candidate target binding proteins. We analyzed the biological functions and molecular signaling pathways of hsa_circ_0000437 in the occurrence and development of RVHD using GO and KEGG bioinformatics. Using GO analysis, we found that system development, positive regulation of the cellular process, regulation of the cellular process, regulation of signaling, positive regulation of the biological process, regulation of cell communication, multicellular organism development, anatomical structure development, localization, and other biological functions may be involved in the development of RVHD in hsa_circ_0000437 (Fig. S1B). KEGG bioinformatics analysis found that the MAPK signaling pathway, the insulin signaling pathway, the rap1 signaling pathway, melanogenesis, the ras signaling pathway, the cAMP signaling pathway, and other signaling pathways may be involved in the occurrence and development of RVHD with hsa_circ_0000437 (Fig. S1C).

### Label-free proteomic mass spectrometry

**Fig. 2:**
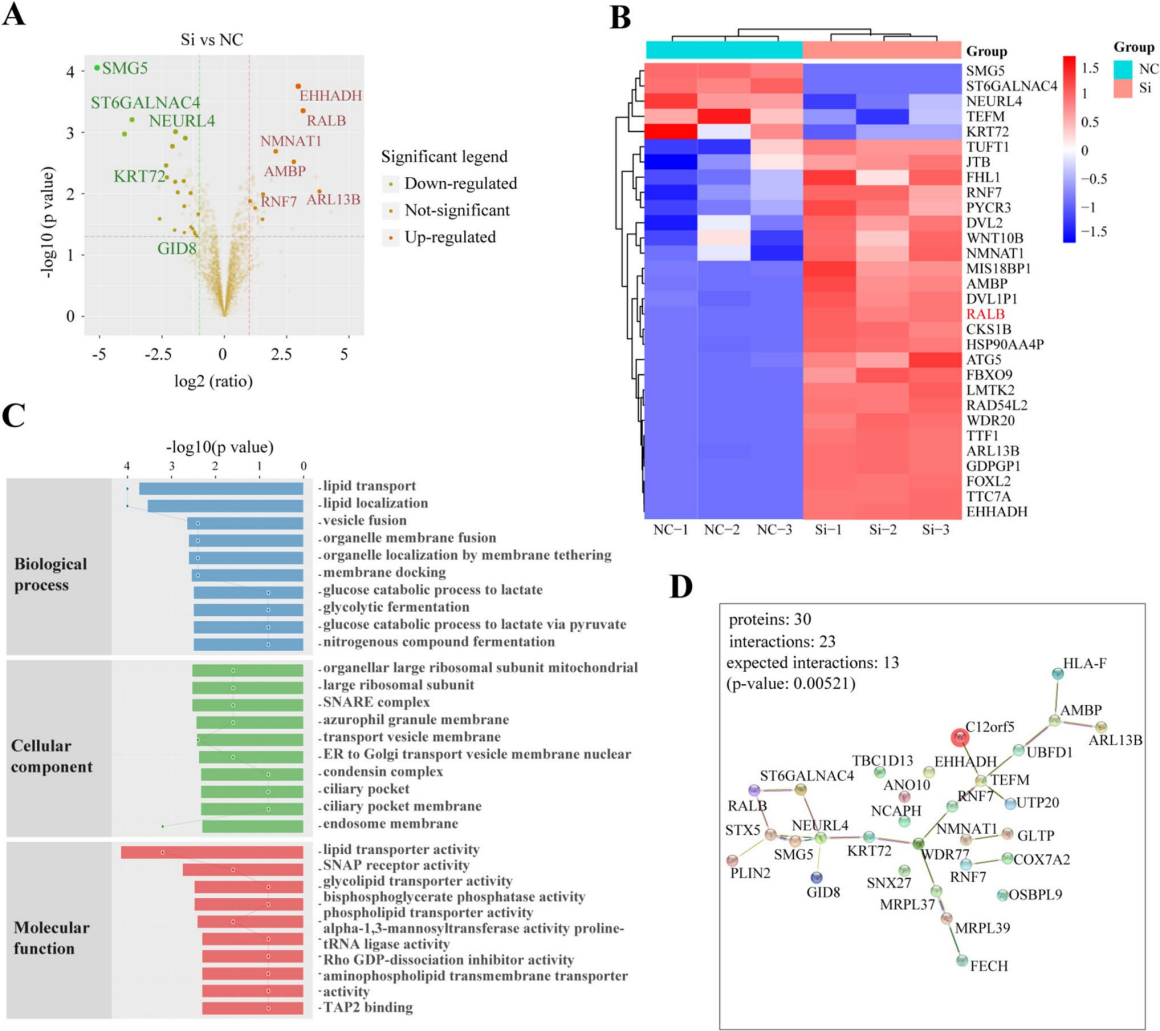
Analysis of label-free proteomic mass spectrometry. **A** Differential protein volcano map is shown. The proteins with significant differences between Si and NC were shown as dark green (downregulated) and orange–red (upregulated), respectively, and non-statistically significant proteins are shown in brown. **B** In differential protein hierarchical cluster analysis, the top color bar represents sample grouping, the bottom is the name of the corresponding sample, and the protein names on the right correspond to the symbol column in the differential protein list. **C** GO enrichment analysis of differentially expressed proteins. The picture shows the top ten GO entries. **D** STRING protein interaction analysis. *n* = 3, *n* stands for replicate samples

Our research found that the abundance of the quantitative dynamic range was within the allowable range of experimental error (Fig. S2A). Quantitative repeatability determines the sensitivity and reliability of quantitative experiments without technical repeatability. Most proteins did not differ in expression, and there was a high consistency among the samples (Fig. S2B). To highlight the significant differentially expressed proteins, we drew a volcano map of differentially expressed proteins (Fig. 2A). Twenty differentially expressed proteins were deviation (EHHADH, RALB, TTC7A, FOXL2, GDPGP1, ARL13B, TTF1, WDR20, RAD54L2, LMTK2, FBXO9, ATG5, HSP90AA4P, CKS1B, DVL1P1, AMBP, MIS18BP1, NMNAT1, WNT10B, DVL2, RNF7, FHL1, JTB, TUFT1 were upregulated, SMG5, ST6GALNAC4, NEURL4, TEFM, and KRT72 were downregulated).

To test the rationality and accuracy of the selected differentially expressed proteins or characteristic differentially expressed proteins, we used the proteins to perform hierarchical clustering analysis and display the samples of each group. In addition to the differentially expressed proteins, proteins with no significant differences were clustered (Fig. 2B). We selected differentially expressed proteins for GO enrichment analysis and analyzed their involvement in biological processes, cellular components, and molecular function. We displayed the first ten GO entries (Fig. 2C).

Analysis of the KEGG signaling pathway revealed no predictive protein-related pathway. A protein interaction network is an essential part of systems biology, which can be used to identify functional genome data and provide an intuitive analysis platform for studying protein structure, function, and evolution. The prediction of the protein interaction network can provide a reference for subsequent mechanism research. We integrated various data using the STRING database and conducted protein interaction analysis (Fig. 2D).

### PRM, RNA pull-down and RIP technology mines targeted binding proteins

**Fig. 3:**
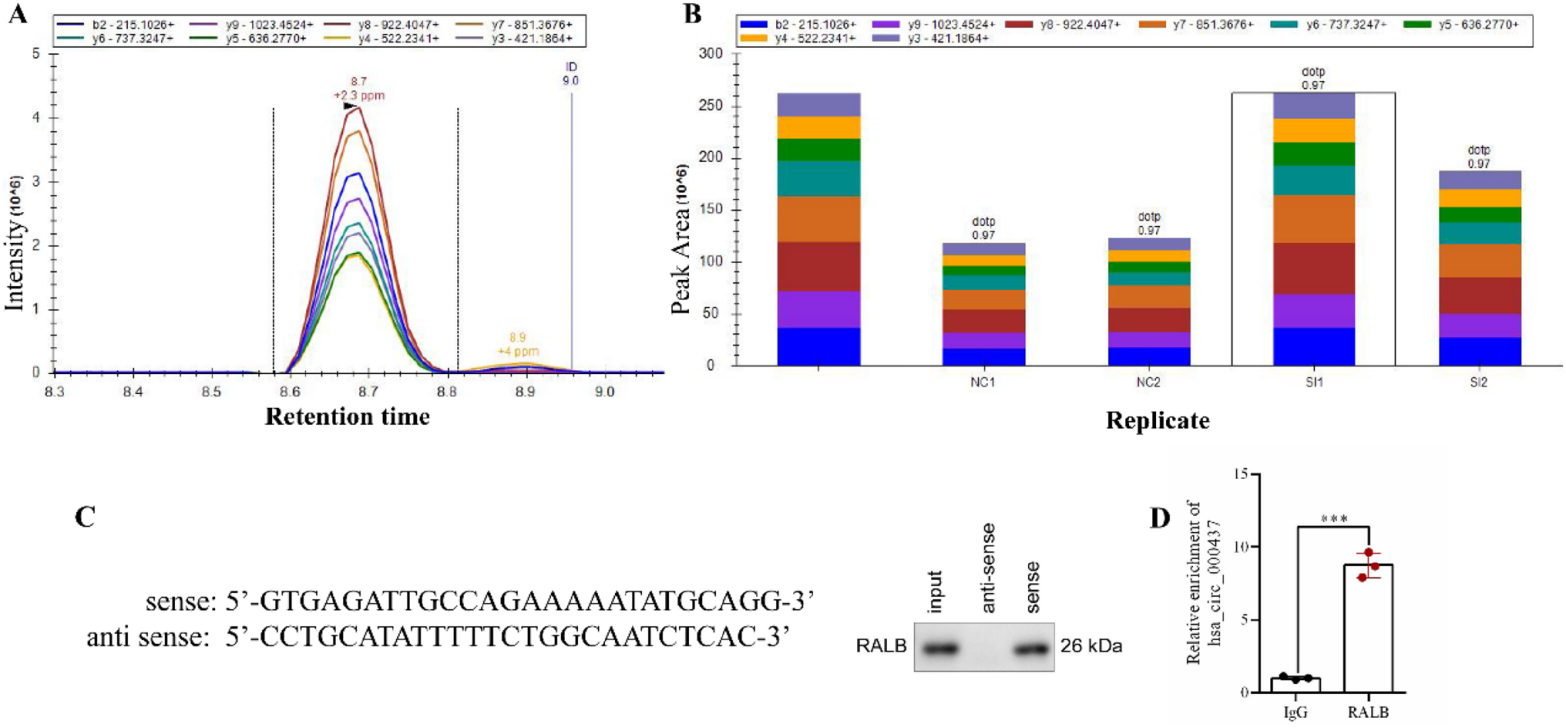
PRM relative quantitative results. **A** Qualitative information on the peptide: the picture shows the qualitative information on the peptide VDCTANTNTCNK. **B** Bar chart of quantitative comparison of polypeptide VDCTANTNTCNK between sample groups. **C** Results of the RNA-pull down assay. **D** Results of the RIP assay.* n* = 3, *n* stands for replicate samples

The absolute quantification of hsa_circ_0000437 enriched RALB was verified using PRM. Different colors represented different fragment ions of the same peptide. These fragment ions have the same chromatographic peak type, indicating that the peptide was identified. The absence of an identical chromatographic peak type indicates that the peptide was not identified. We performed qualitative (Fig. 3A) and quantitative (Fig. 3B) analyses of these peptides. This specific peptide segment has the highest peak area among Si-1, Si-2 and Si-3 (experimental groups) compared with NC-1, NC-2 and NC-3 (control groups) (Fig. 3B). We also explored whether hsa_circ_0000437 directly interacts with RALB through RNA pull-down experiments. The results showed that hsa_circ_0000437 was significantly enriched in the RALB group compared with the RALB-NC group (Fig. 3C). The RIP results showed that, compared with the IgG group, hsa_circ_0000437 was significantly enriched in the RALB group (Fig. 3D). Combined with label-free proteomics, PRM, RNA pull-down assay and RIP assay indicates that hsa_circ_0000437 can direct bind to RALB.

### The direct and binding regulatory relationship between hsa_circ_0000437 and let-7f-5p, as well as let-7f-5p and RALB

**Fig. 4:**
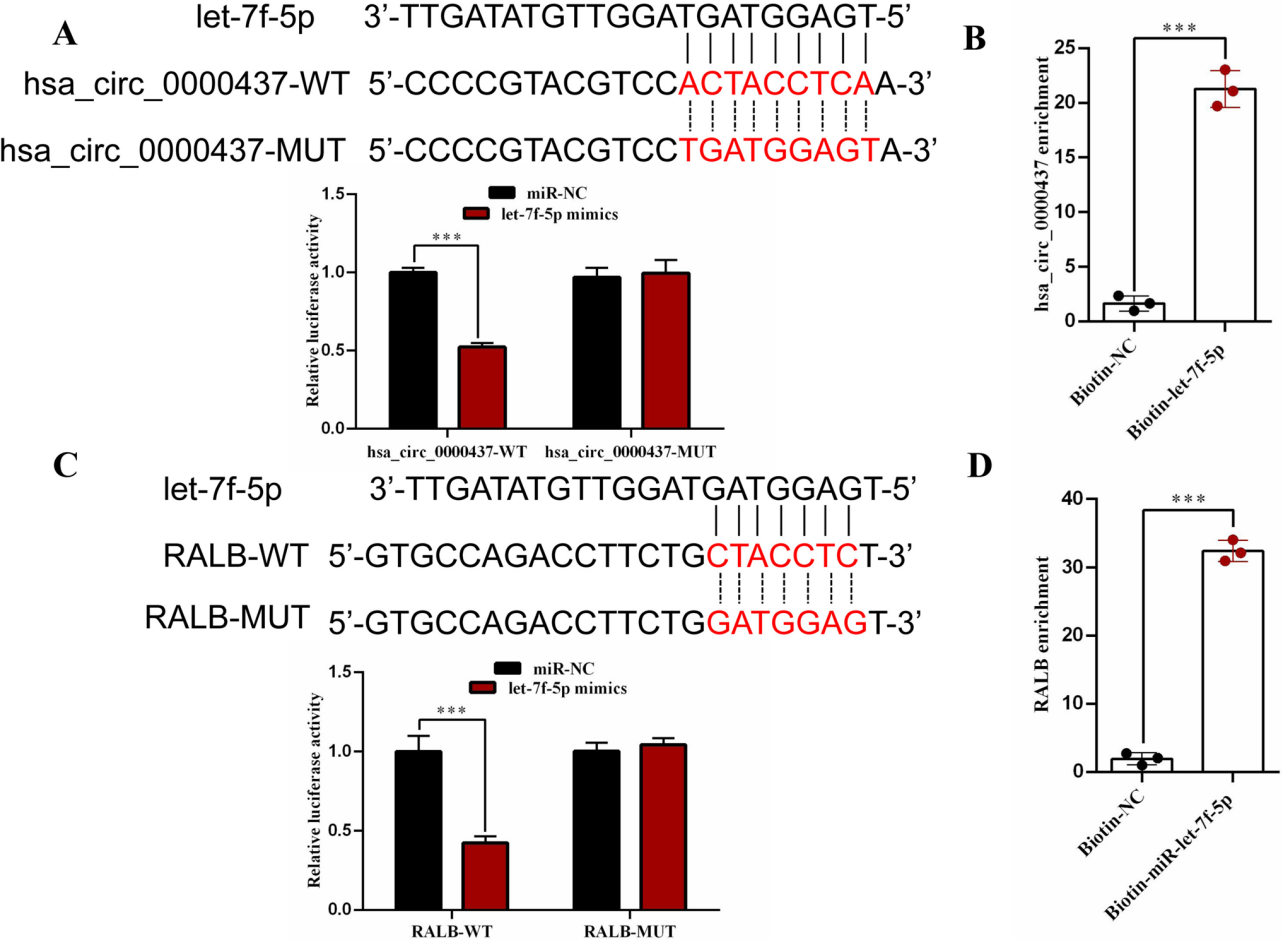
**A** Dual-luciferase reporter assay confirmed the presence of binding sites between hsa_circ_0000437 and let-7f-5p. **B** RNA pull-down assay confirmed the binding between hsa_circ_0000437 and let-7f-5p. **C** Dual-luciferase reporter assay confirmed the presence of binding sites between let-7f-5p and RALB. **D** RNA pull-down assay confirmed the binding between let-7f-5p and RALB. *n* = 3, *n* stands for biological replicates

We previously predicted that hsa_circ_0000437 might be combined with let-7f-5p using Circbank and circular RNA interaction software [21]. To verify this prediction, we constructed a dual-luciferase reporting system. The results showed that when co-transfected with wild type (WT) and NC or miRNA, the mimic of let-7f-5p significantly reduced luciferase activity. Subsequently, we cloned a mutant sequence into the 3’ untranslated region (UTR) of the psiCHECK2 plasmid, the binding site in hsa_circ_0000437. However, after co-transfection with mutation (Mut) and corresponding miRNA mimic, we did not observe significant changes in luciferase activity (Fig. 4A). In addition, we conducted RNA pull-down experiments to investigate whether hsa_circ_0000437 directly interacts with let-7f-5p, and the results showed that let-7f-5p was significantly enriched in the hsa_circ_0000437 group compared to the antisense sequence group (Fig. 4B). The above evidence reflects hsa_circ_0000437 direct and binding let-7f-5p.

We have confirmed that hsa_circ_0000437 can directly bind to RALB. To explore whether there is a binding between let-7f-5p and RALB, we first conducted a dual-luciferase reporter gene test. We constructed a dual-luciferase reporting system by inserting the sequence of RALB into the 3 ‘UTR of the psiCHECK2 plasmid. The results showed that let-7f-5p mimic significantly reduced luciferase activity when co-transfected with WT, NC, or miRNA. Subsequently, we cloned a mutant sequence into the 3 ‘UTR of the psiCHECK2 plasmid, a binding site in RALB. However, no significant changes in luciferase activity were observed after co-transfection with Mut and corresponding miRNA mimics (Fig. 4C). We also explored whether RALB directly interacts with let-7f-5p through RNA pull-down experiments. The results showed that RALB was significantly enriched in the let-7f-5p group compared with the miR-NC group (Fig. 4D). This indicates that RALB can also direct and bind let-7f-5p.

### Diagnostic value of hsa_circ_0000437, let-7f-5p, and RALB in RVHD

**Fig. 5:**
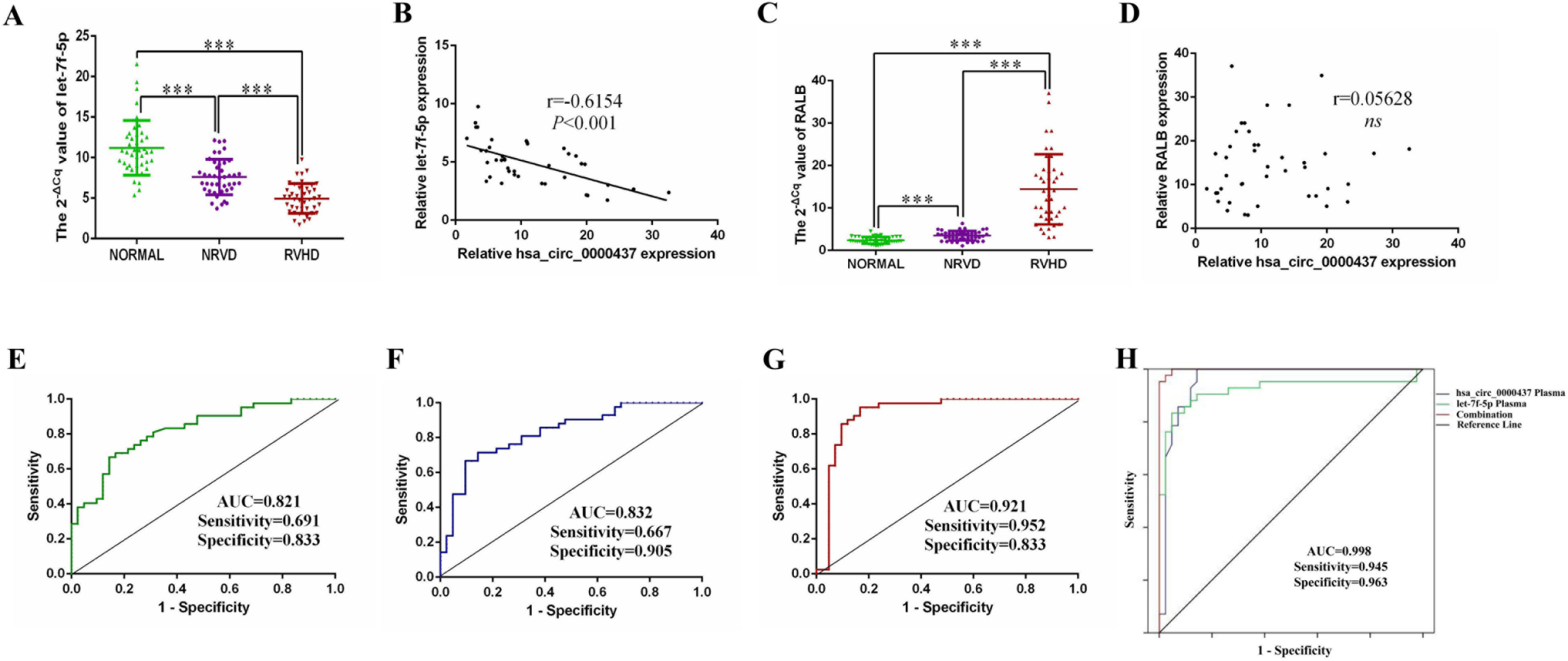
Expression levels of let-7f-5p and RALB in RVHD plasma samples and diagnostic value of let-7f-5p and hsa_circ_0000437 combined with let-7f-5p. **A** Expression level of let-7f-5p in the RVHD group was significantly lower than that in the NRVD group and control group, and the NRVD group was also significantly lower than that in the control group (*n* = 42). **B** Pearson correlation analysis showed that hsa_circ_0000437 was negatively correlated with let-7f-5p expression in 42 RVHD plasma samples. **C** Expression level of RALB in the RVHD group was significantly higher than that in the NRVD group and control group, and the NRVD group was also significantly higher than that in the control group (*n* = 42). **D** Pearson correlation analysis showed that hsa_circ_0000437 was not correlated with RALB expression in 42 RVHD plasma samples. **E** AUC of let-7f-5p in RVHD compared with the NRVD group. **F** AUC of let-7f-5p in NRVD compared with the control group. **G** AUC of let-7f-5p in RVHD compared with the control group. **H** AUC after the combination of hsa_circ_0000437 and let-7f-5p in RVHD. AUC: the area under the curve, *n* stands for replicate samples, ***P < 0.001, *ns*: no significance

We measured expression levels of let-7f-5p in 42 RVHD patients, 42 NRVD patients, and healthy controls and found that let-7f-5p was significantly lower in the RVHD group than in the NRVD and control groups, let-7f-5p was also lower expressed in the NRVD groups compared to the control groups (Fig. 5A). This finding was contrary to the expression trend of hsa_circ_0000437 found earlier [21]. Correlation analysis showed that expression levels of hsa_circ_0000437 and let-7f-5p in RVHD patients were significantly negatively correlated (Fig. 5B). We also examined the expression level of RALB in clinical samples. We found that RALB was significantly higher in the RVHD group than in the NRVD and control groups; RALB was also highly expressed in the NRVD groups compared to the control groups (Fig. 5C). However, correlation analysis showed that expression levels of hsa_circ_0000437 and RALB in RVHD patients were no significantly correlated (Fig. 5D). To clarify the diagnostic value of let-7f-5p in RVHD, a receiver operating characteristic curve (ROC) was plotted. The diagnostic value of let-7f-5p in RVHD was evaluated using the area under the curve (AUC). The AUC of the RVHD group was 0.821 compared with the NRVD group. The sensitivity and specificity were 0.691 and 0.833, respectively (Fig. 5E). The AUC of the NRVD group was 0.832 compared with the control group, and the sensitivity and specificity were 0.667 and 0.905, respectively (Fig. 5F). The AUC of RVHD group was 0.921 compared with the control group, the sensitivity, and specificity were 0.952 and 0.833, respectively (Fig. 5G). These findings suggest that let-7f-5p has diagnostic value in RVHD, combined with our previously verified AUC of hsa_circ_0000437 in the RVHD and the control groups.

The combined ROC curve analysis showed that the AUC of the RVHD group was 0.998 compared with the control group after the combined analysis of hsa_circ_0000437 and let-7f-5p in RVHD plasma, and the sensitivity and specificity were 0.945 and 0.963, respectively (Fig. 5H), suggesting excellent diagnostic value.

### Hsa_circ_0000437 sponge absorption of let-7f-5p affects the biological function of RVHD progression

**Fig. 6:**
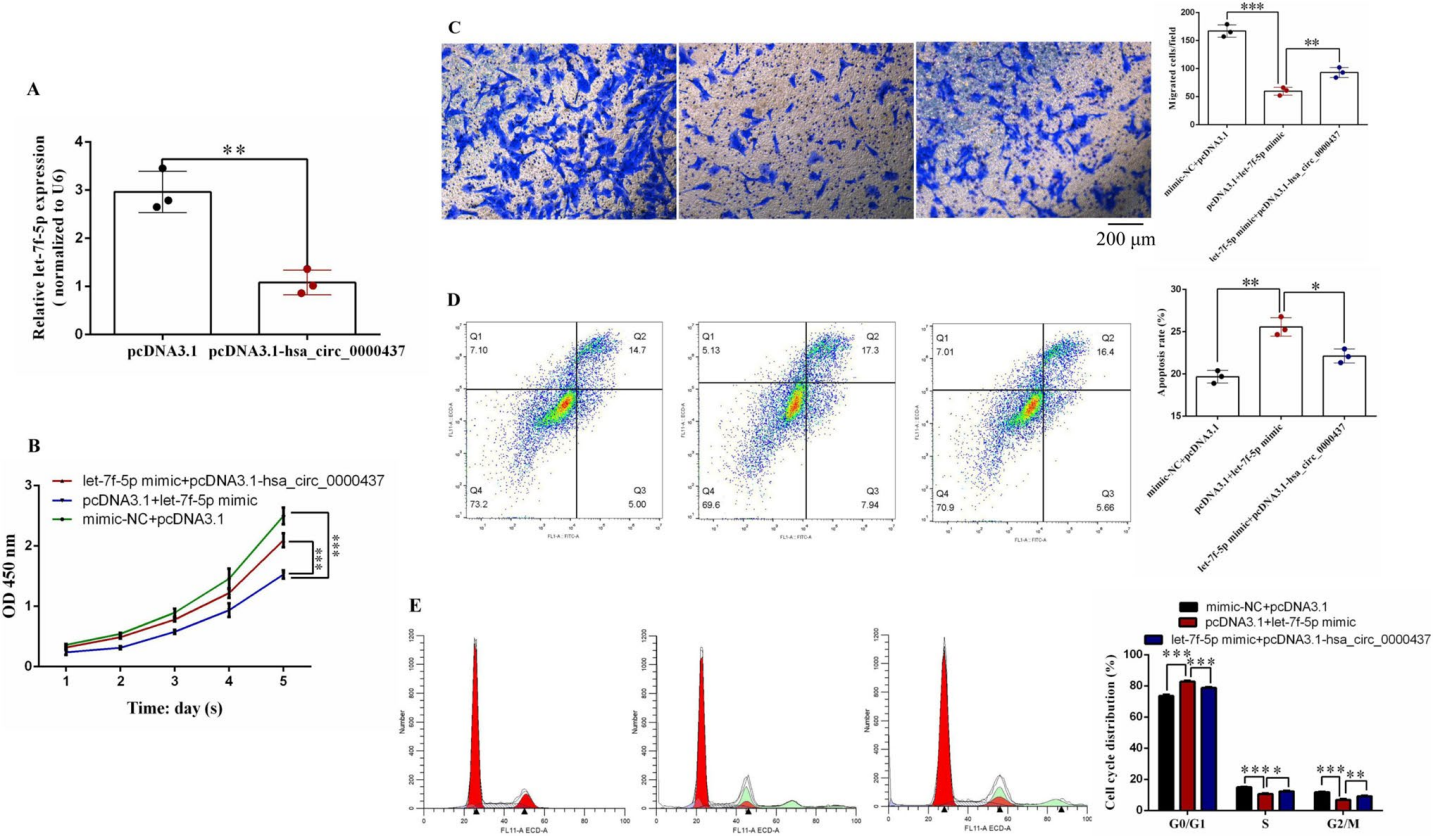
Inhibition of let-7f-5p by hsa_circ_0000437 sponge after absorption affects related biological functions of hVICs cells. **A** Relative expression level of let-7f-5p after overexpression of hsa_circ_0000437. **B** Rescue effect of cell proliferation after simultaneous overexpression of hsa_circ_0000437 and let-7f-5p in hVICs cells. **C** Rescue effect of cell migration after simultaneous overexpression of hsa_circ_0000437 and let-7f-5p in hVICs cells. Scale bar = 200 μm. **D** Rescue effect of apoptosis after simultaneous overexpression of hsa_circ_0000437 and let-7f-5p in hVICs cells. **E** Rescue effect of the cell cycle after simultaneous overexpression of hsa_circ_0000437 and let-7f-5p in hVICs cells. *n* = 3, *n* stands for biological replicates

After overexpression of hsa_circ_0000437, we found that the let-7f-5p expression level was significantly downregulated in hVICs cells (Fig. 6A).

We also investigated the biological functions related to the influence of let-7f-5p absorption by hsa_circ_0000437 sponge on RVHD progression using rescue experiments. Overexpression of hsa_circ_0000437 alleviated the growth inhibition effect of let-7f-5p in hVICs cells (Fig. 6B). We found that hsa_circ_0000437 mitigated the inhibition of migration ability caused by let-7f-5p (Fig. 6C). Flow cytometry showed that hsa_circ_0000437 restored the apoptosis-promoting effect of let-7f-5p on hVICs cells (Fig. 6D). Overexpression of hsa_circ_0000437 caused let-7f-5p to affect the cell cycle distribution of hVICs, which changed from being blocked in the G0/G1 phase to the G2/M phase (Fig. 6E). These findings suggest that hsa_circ_0000437 sponge adsorbs let-7f-5p to regulate hVICs cell-related phenotypes, promoting cell proliferation, migration, and cycle progression and inhibiting apoptosis.

### Hsa_circ_0000437 regulates MAPK signaling pathway to promote RVHD process after targeting RALB

**Fig. 7:**
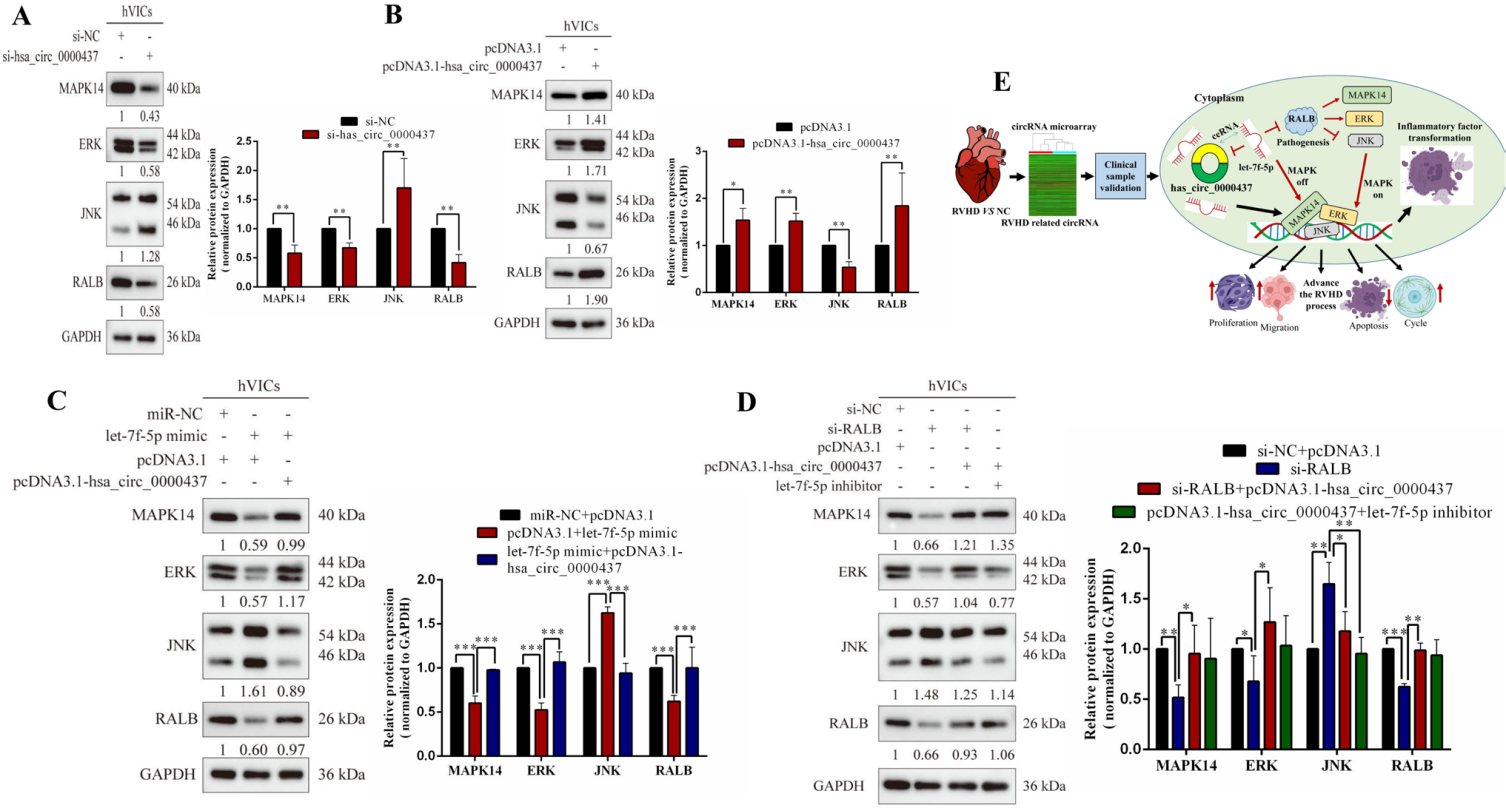
hsa_circ_0000437/let-7f-5p/RALB axis promotes inflammatory transformation during RVHD progression by activating the MAPK signaling pathway. Western blotting assay of MAPK14, ERK, JNK, and RALB proteins in hVICs cells transfected with si-hsa_circ_0000437 (**A**), pcDNA3.1-hsa_circ_0000437 (**B**), let-7f-5p mimic and pcDNA3.1-hsa_circ_0000437 (**C**), si-RALB, pcDNA3.1-hsa_circ_0000437 and let-7f-5p inhibitor (**D**). Numbers indicate the relative expression of proteins, and GAPDH serves as an internal reference. **E** Diagram of the regulatory mechanism of hsa_circ_0000437/let-7f-5p/RALB axis promoting the occurrence and development of RVHD, the target hsa_circ_0000437 was identified by microarray and confirmed in the cytoplasm that RALB could promote inflammatory transformation during RVHD by activating MAPK signaling pathway. RALB was also inhibited by let-7f-5p and enhanced by ceRNA hsa_circ_0000437 (some elements were created using BioRender.com).* n* = 3, *n* stands for biological replicates

After silencing and overexpressing hsa_circ_0000437, we performed Western blotting and found that RALB expression levels decreased (Fig. 7A) and increased (Fig. 7B), respectively. MAPK14, ERK, and JNK are the primary regulatory factors in the MAPK signaling pathway, in which MAPK14 and ERK positively regulate it, and JNK negatively regulates it [24]. After silencing hsa_circ_0000437, the expression levels of MAPK14 and ERK were significantly decreased, while the expression levels of JNK were significantly increased (Fig. 7A). After overexpression of hsa_circ_0000437, levels MAPK14, ERK, and JNK were reversed to those of the silencing experiments (Fig. 7B).

After overexpression of let-7f-5p, expression levels of RALB decreased, and MAPK14 and ERK changed in parallel with RALB; however, JNK significantly increased (Fig. 7C). After hsa_circ_0000437 overexpression, the decreased expression levels of RALB, MAPK14, and ERK were significantly reversed, while the increased expression levels of JNK were inhibited (Fig. 7C).

We also analyzed the pathways and molecular mechanisms influencing RVHD after hsa_circ_0000437 targets RALB. After silencing RALB, protein expression significantly decreased; MAPK14 and ERK also significantly decreased, while JNK significantly increased (Fig. 7D). When hsa_circ_0000437 expression level was overexpressed simultaneously, MAPK signaling pathway-related proteins and RALB levels were significantly higher than with RALB silenced alone (Fig. 7D). MAPK14, ERK, and RALB significantly increased; in contrast, JNK significantly decreased after hsa_circ_0000437 was overexpressed and let-7f-5p was silenced (Fig. 7D). These findings suggest that hsa_circ_0000437 regulates the MAPK signaling pathway and promotes RVHD after sponge adsorption of let-7f-5p by targeting RALB. This process promotes inflammatory transformation and functional biological processes in RVHD (Fig. 7E).

## Discussion

RVHD morbidity and mortality have decreased worldwide; nevertheless, it still threatens human health, and there is a long way to go to identify targets for early intervention and diagnosis [25]. Several lines of evidence suggest that the abnormal expression of circRNAs is associated with RVHD progression [21, 26, 27]. Hu et al. revealed the circRNA expression profile involved in rheumatic heart disease-associated atrial fibrillation using high-throughput sequencing technology, which may lay a foundation for diagnosing and treating this disease and its mechanism of action [26]. Similarly, for atrial fibrillation associated with valvular heart disease, researchers constructed circRNA differential expression profiles using RNA-seq and conducted clinical sample verification and GO and KEGG bioinformatics analysis, which laid a foundation for future circRNA-related research [28].

There are also studies on the relationship between circRNA and RVHD. Zhang et al. found that the has_circ_0003748 sponge acted on RVHD to promote its progression after absorbing miR-577 [27]. Our previous study found that hsa_circ_0000437 was highly expressed in RVHD plasma clinical samples, and hsa_circ_0000437 participated in the biological functions related to RVHD progression [21]. We identified sponge miRNA let-7f-5p and explored related biological functions through circRNA microarray results with bioinformatics prediction. RALB binding to hsa_circ_0000437 was identified using label-free mass spectrometry and PRM combined with RNA-pull down and RIP assay. We clarified the biological function and pathogenesis of hsa_circ_0000437 promoting RVHD progression.

Wu et al. analyzed the circRNA microarray results and found that 13 circRNAs were downregulated and 46 were upregulated in patients with hypertension; four upregulated circRNAs included hsa_circ_0000437 [29]. Another study found significant differences in hsa_circ_0000437 expression between gastric cancer and paracancer tissues; a mechanism study revealed that hsa_circ_0000437 targets serine/arginine-rich splicing factor 3 and programmed cell death 4 to regulate the proliferation, invasion, migration, and apoptosis of gastric cancer cells. It also confirmed that hsa_circ_0000437 promoted tumor growth through animal experiments [30]. Zhong et al. identified differentially expressed autophagy-related circRNAs in 144 prostate cancer patients using Pearson correlation analysis and constructed a prognostic model based on five autophagy-related circRNAs including hsa_circ_0000437 using correlation algorithm and regression analysis [31]. These findings demonstrate that hsa_circ_0000437 is differentially expressed in cardiovascular diseases and tumors. circRNA and miRNA interact with one another in a ceRNA-like manner during disease progression, primarily because of miRNA response elements similar to mRNA in circRNA, which can competitively bind sponge miRNA to regulate mRNA expression levels, thus regulating disease progression [32, 33]. When ceRNA is silenced, mRNA degrades in miRNA-containing RNA-induced silencing complexes. When ceRNA is activated, it competitively binds to that complex, reducing the inhibitory function of miRNA and upregulating target genes [34, 35].

This study showed that let-7f-5p expression in RVHD was opposite to that of hsa_circ_0000437 (Fig. 5A). Levels of let-7f-5p and hsa_circ_0000437 were negatively correlated (Fig. 5B). However, the expression of RALB was opposite to that of let-7f-5p (Fig. 5C) and had no correlation with hsa_circ_0000437 (Fig. 5D). The diagnostic value of let-7f-5p and hsa_circ_0000437 were evaluated using AUC. Let-7f-5p and hsa_circ_0000437 emerged as strong candidates for non-invasive diagnostic markers for RVHD (Fig. 5E–H) [21].

After let-7f-5p overexpression, hVICs growth, metastasis, cycle inhibition, and pro-apoptosis were recovered by hsa_circ_0000437 overexpression (Fig. 6B–E). These findings suggest that after the absorption of let-7f-5p by the hsa_circ_0000437 sponge, both promote the RVHD process via ceRNA coexistence. RALB of hsa_circ_0000437 was extracted using label-free proteomic mass spectrometry and PRM, RNA pull-down and RIP (Figs. 2,3). Hsa_circ_0000437, let-7f-5p, and RALB formed a ceRNA regulatory network to regulate RVHD occurrence and progression.

Regarding let-7f-5p and cardiovascular disease, a study found that let-7f-5p was expressed at significantly higher levels in plasma samples from a spontaneous coronary artery dissection group than in an atherothrombotic acute myocardial infarction group [36]. Cecconi et al. found that acute ST-segment elevation myocardial infarction was also associated with increased let-7f-5p expression [37]. Wu et al. found that the serum expression level of let-7f-5p in children with dilated cardiomyopathy was significantly higher than in the control group [38]. In other diseases, exosome-derived let-7f-5p activated DUSP1/Erk1/2 signal transduction, promoting angiogenesis in endothelial cells [39]. Using qRT–PCR expression detection, Niculae et al. found that let-7f-5p was less expressed in colorectal cancer tissues than in paraneocarcinoid tissues [40]. Let-7f-5p was significantly less expressed in a bacteria-induced pulmonary fibrosis rat model and negatively regulated the PI3K/AKT/COX2 signaling pathway [41]. Li et al. found that IL-1β reduced wound healing by activating the NF-κB signaling pathway and increasing let-7f-5p expression. This process reduced IGF-1 in dendritic epidermal T cells, suggesting that let-7f-5p could be used in wound healing [42]. These findings suggest that let-7f-5p may also play an essential role in RVHD progression.

RALB regulates cell growth, tumor metastasis, and granule secretion [43, 44]. There have been advances in RALB in cardiovascular disease; for example, RALB is required for autophagy in mTOR-dependent cardiomyocytes, and RALB-associated RalGDS-mediated autophagy induction and exocyst function is critical for load-induced cardiac hypertrophy [45]. Wersall et al. found that Ral GTPases, including RALB, may be targeted for selective regulation of inflammatory diseases and platelet-mediated immune cell recruitment [46]. Serine–threonine kinase 38 and RALB assist in regulating autophagy and apoptosis events during autophagy induction [47]. Zhang et al. found that RALB may be an immune-related regulatory gene affecting patients with depression, which can provide a novel direction for diagnosing and treating patients with major depression [48]. Wang et al. found that RALB may be an essential regulator of lumen formation in epithelial tissues through functional analysis of candidate genes [49]. Other studies found that RALB is indispensable in tumor occurrence and progression [50]. These findings suggest that hsa_circ_0000437-targeted binding of RALB in this study may also be indispensable in the progression of RVHD.

The MAPK signaling pathway is a common intersection pathway involved in cell proliferation, inflammation, apoptosis, and other related signal transduction pathways. It transmits extracellular signals to intracellular signals and participates in various cell activities [51, 52]. Angiotensin-(1–7) may inhibit the MAPK and NF-κB signaling pathways, delaying inflammatory joint damage and myocardial fibrosis and activating the downstream TGF-/Smad pathway [53]. Zhao et al. found that TGF-β1 secreted by CD4 + T cells participates in valve fibrosis during RVHD via the MAPK pathway [54]. Hepatocyte growth factor and basic fibroblast growth factor regulate atrial fibrosis progression in RVHD patients via the MAPK signaling pathway [55]. Garmany et al. found that RAS–MAPK grade pathway activation may participate in hypertrophic cardiomyopathy [56]. After quantitative phosphoproteomics analysis, MAPK pathway expression of ischemic heart failure was upregulated [57]. The calcineurin/NFAT and MAPK/ERK signaling pathways are interdependent signal cascade networks participating in myocardial hypertrophy [58]. These findings suggest that the MAPK signaling pathway will likely participate in RVHD progression along the hsa_circ_0000437/let-7f-5p/RALB axis.

We found that the hsa_circ_0000437/let-7f-5p/RALB axis promotes the transformation of inflammatory cytokines in RVHD by activating the MAPK signaling pathway (Fig. 7). However, we have not yet constructed an animal model to explore the role of hsa_circ_0000437 in the progression of RVHD in vivo; nevertheless, the phenomenon lays the foundation for studying the role of hsa_circ_0000437 in RVHD.

## Conclusions

We found that the hsa_circ_0000437/let-7f-5p/RALB axis (a set of ceRNA regulatory systems) promotes inflammatory and related biological function progression in RVHD progression after activation of the MAPK signaling pathway. Our findings might provide new perspectives on RVHD diagnosis and treatment.

## Supplementary Information

Below is the link to the electronic supplementary material.

Supplementary file1 (XLS 62 KB)

Supplementary file2 (DOCX 1729 KB)

Supplementary file3 (PDF 28017 KB)

Supplementary file4 (DOCX 16 KB)

## Data Availability

The authors declare that the data supporting the findings of this study are available in the article. Extra data or information are available from the corresponding authors upon reasonable request.

## Abbreviations

circRNAs: Circular RNAs
RVHD: Rheumatic valvular heart disease
hVICs: Human valve interstitium cells
RALB: RAS-like proto-oncogene B
MAPK: Mitogen-activated protein kinase
ceRNAs: Competing endogenous RNAs
PRM: Parallel reaction monitoring
GO: Gene ontology
KEGG: Kyoto Encyclopedia of Genes and Genomes
NRVD: Non-rheumatic valvular disease
ROC: Receiver operating characteristic curve
AUC: The area under the curve
