# Supplementary material for: Hsa_circ_0000437 promotes the progression of rheumatic valvular heart disease by activating the mitogen-activated protein kinase signaling pathways after sponging let-7f-5p and targeting RAS-like proto-oncogene B
Source: Hum Cell. 2025 Dec 15;39(1):19. doi: 10.1007/s13577-025-01331-7 (PMC12705832; doi:10.1007/s13577-025-01331-7)
Supplement: Supplementary file 2 — Supplementary file2 (DOCX 1729 KB) [file 13577_2025_1331_MOESM2_ESM.docx]

**
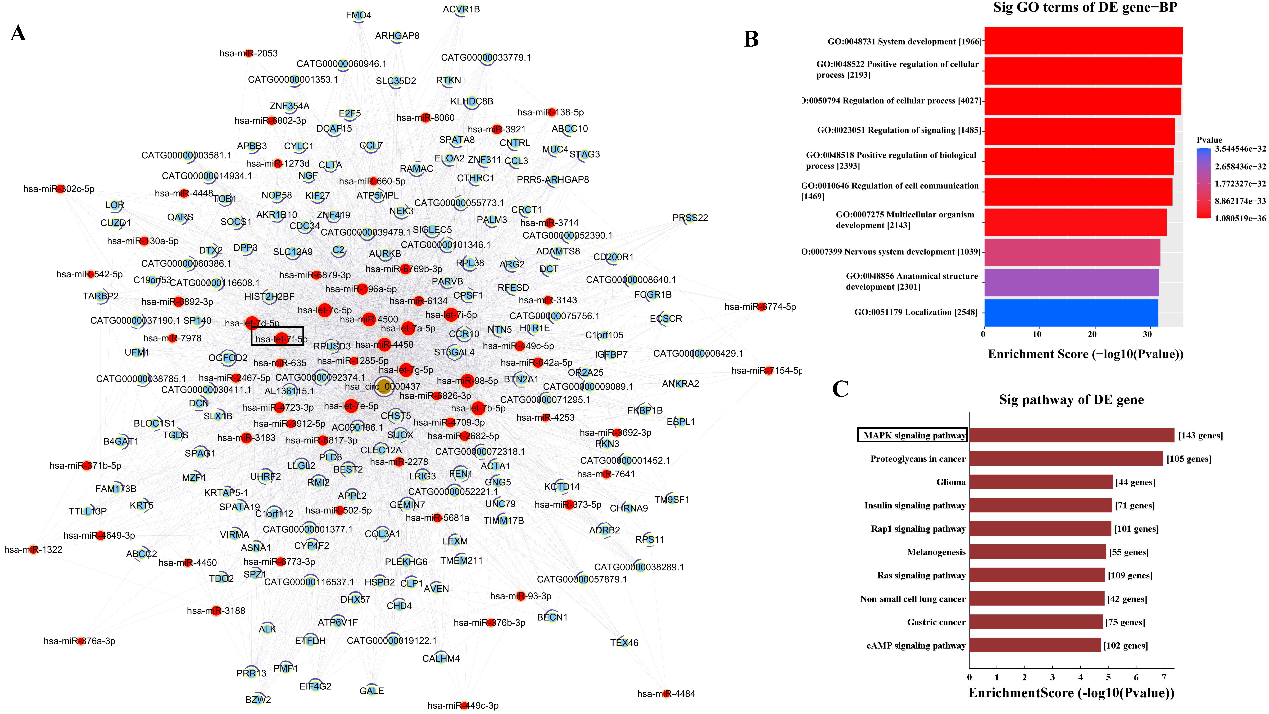
**

**Supplementary** **Fig S1.** Bioinformatics analysis explored the biological functions and molecular mechanisms of hsa_circ_0000437 in RVHD. (A) Bioinformatics analysis of the ceRNA network of hsa_circ_0000437, let-7f-5p is one of the ceRNA. (B) GO analysis of hsa_circ_0000437 related biological functions. (C) KEGG analysis of hsa_circ_0000437 related molecular signaling pathway. MAPK is one of the signaling pathways.


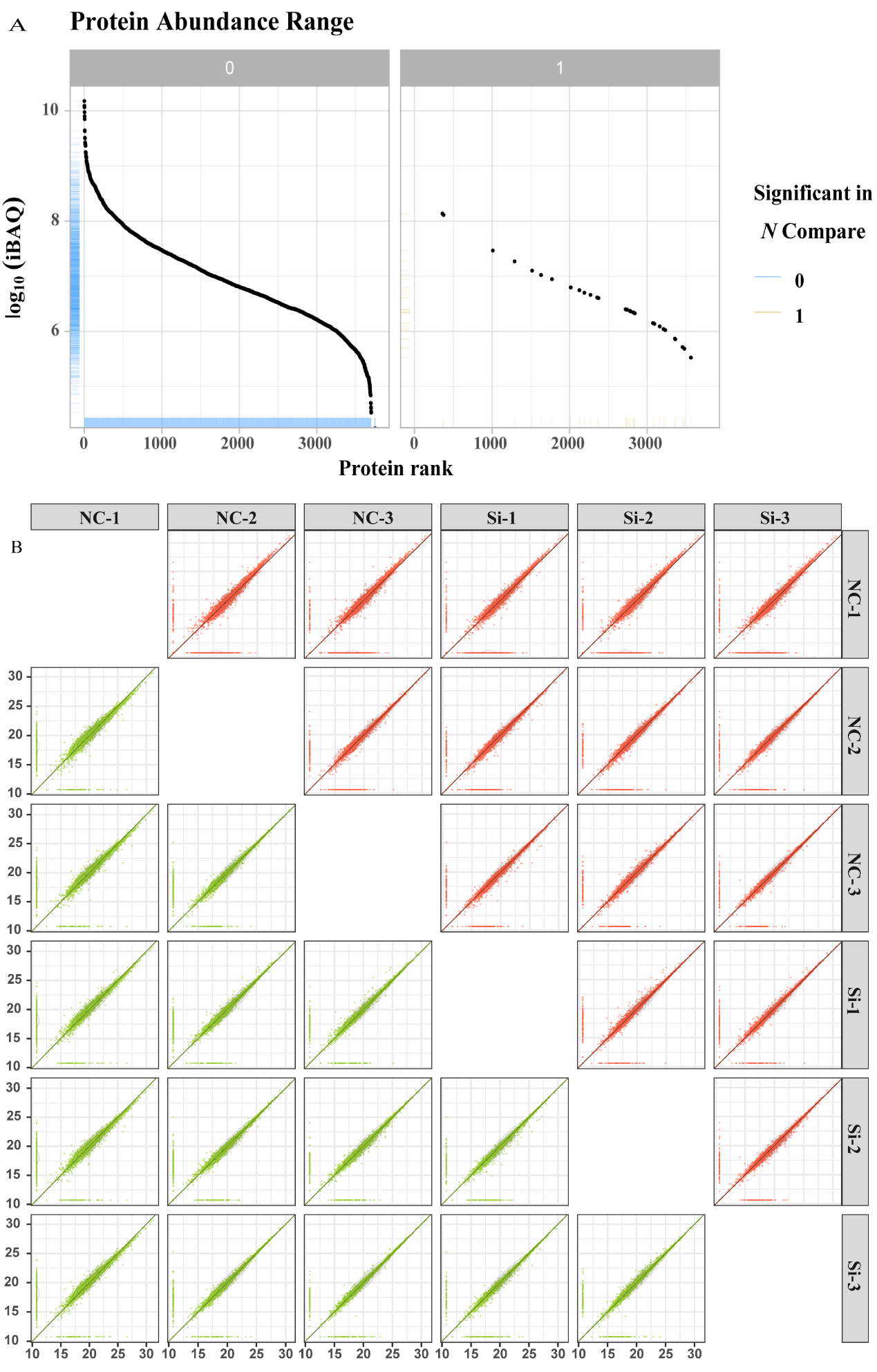


**Supplementary Fig S2.** Analysis of label-free proteomic mass spectrometry quality inspection result. (A) The protein abundance distribution map, the quantitative dynamic range of more than six orders of magnitude, the y-axis represents the absolute amount of protein, and different colors indicate whether the protein is differentially expressed. (B) Quantitative repeatability analysis between experiment and control samples (red is normalized data, green is raw data).
