# Supplementary figures and images for: Hsa_circ_0000437 promotes the progression of rheumatic valvular heart disease by activating the mitogen-activated protein kinase signaling pathways after sponging let-7f-5p and targeting RAS-like proto-oncogene B
Source: Hum Cell. 2025 Dec 15;39(1):19. doi: 10.1007/s13577-025-01331-7 (PMC12705832; doi:10.1007/s13577-025-01331-7)

**Fig. 3C**

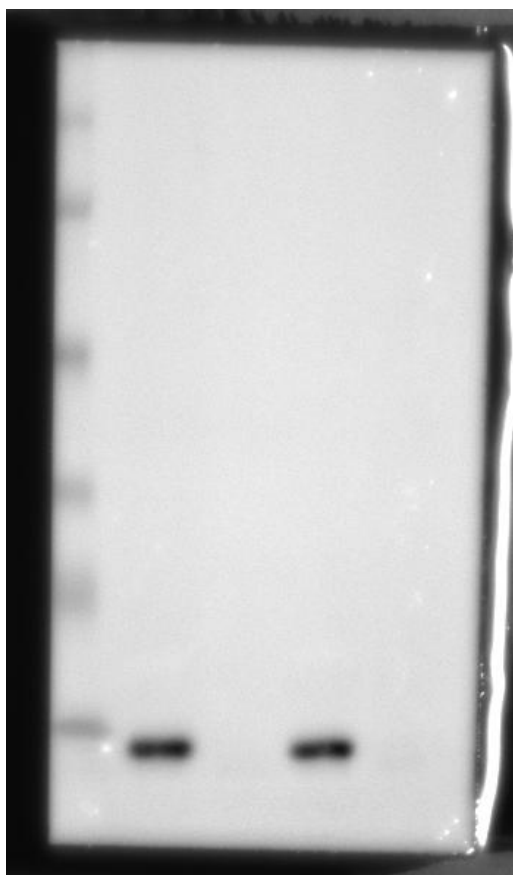

**Fig. 7A**

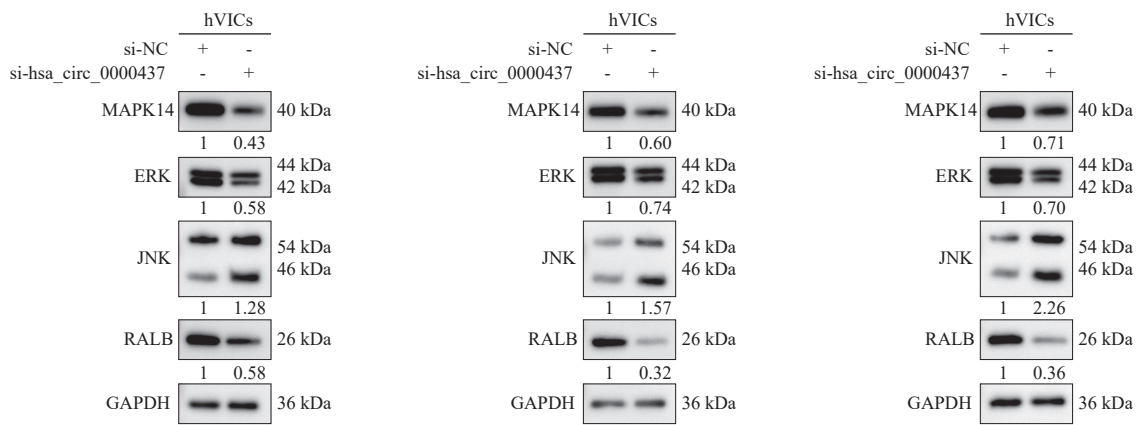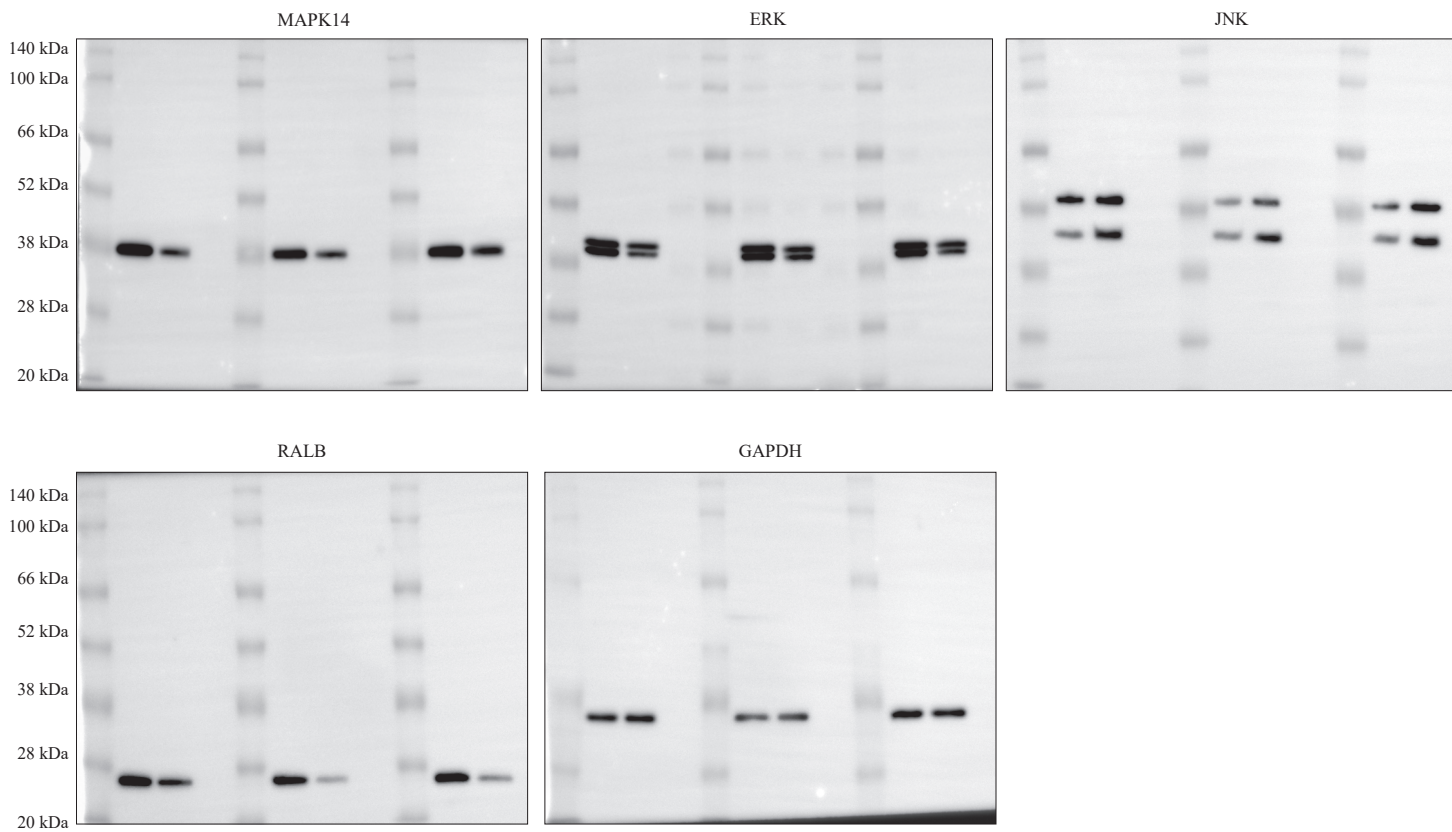

Fig. 7B

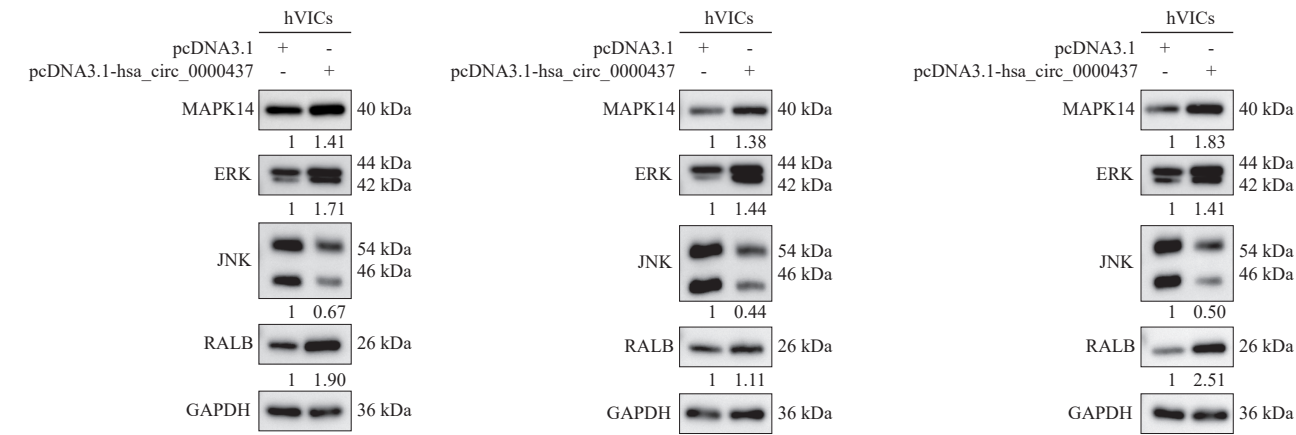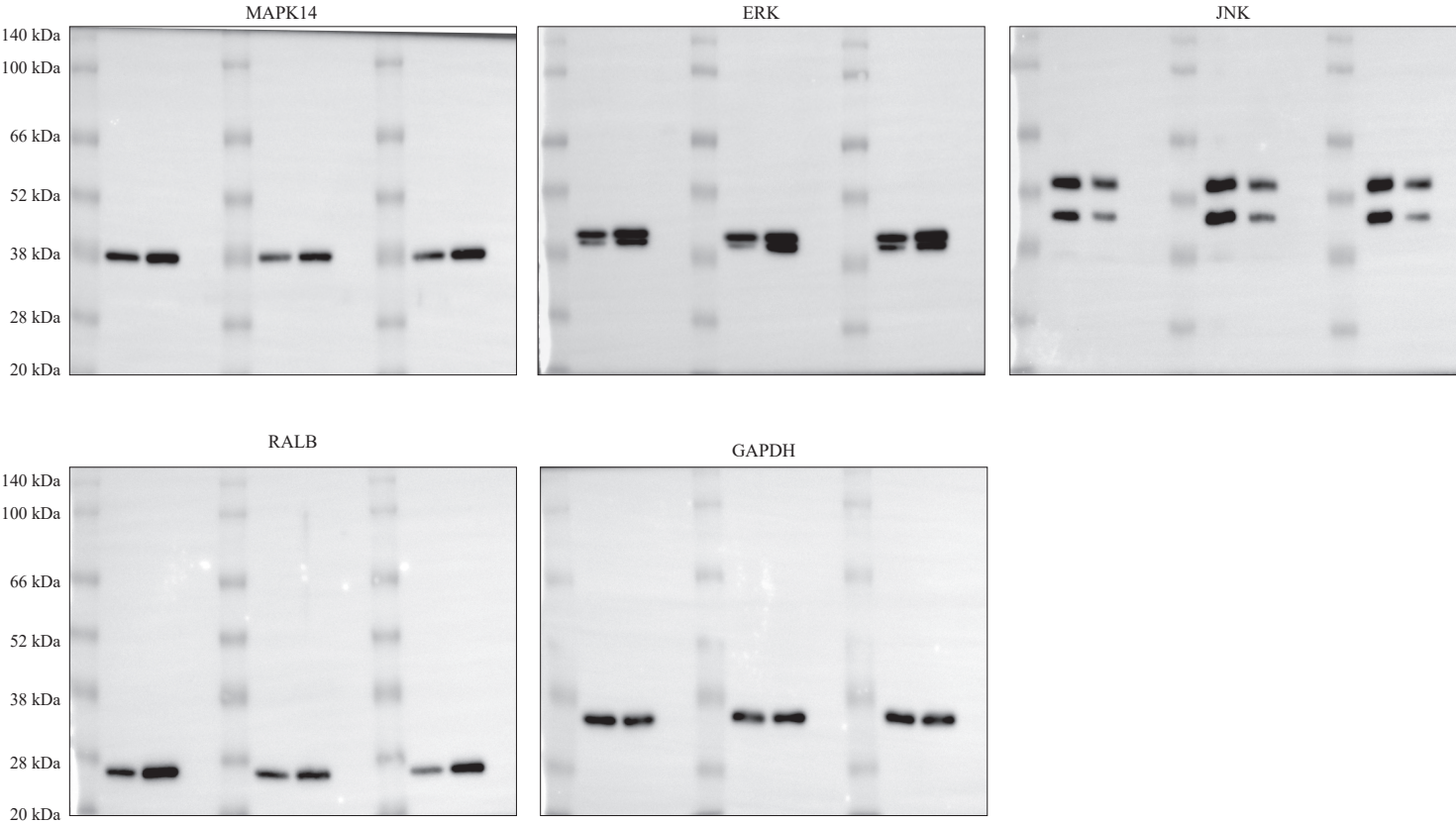

Fig. 7C

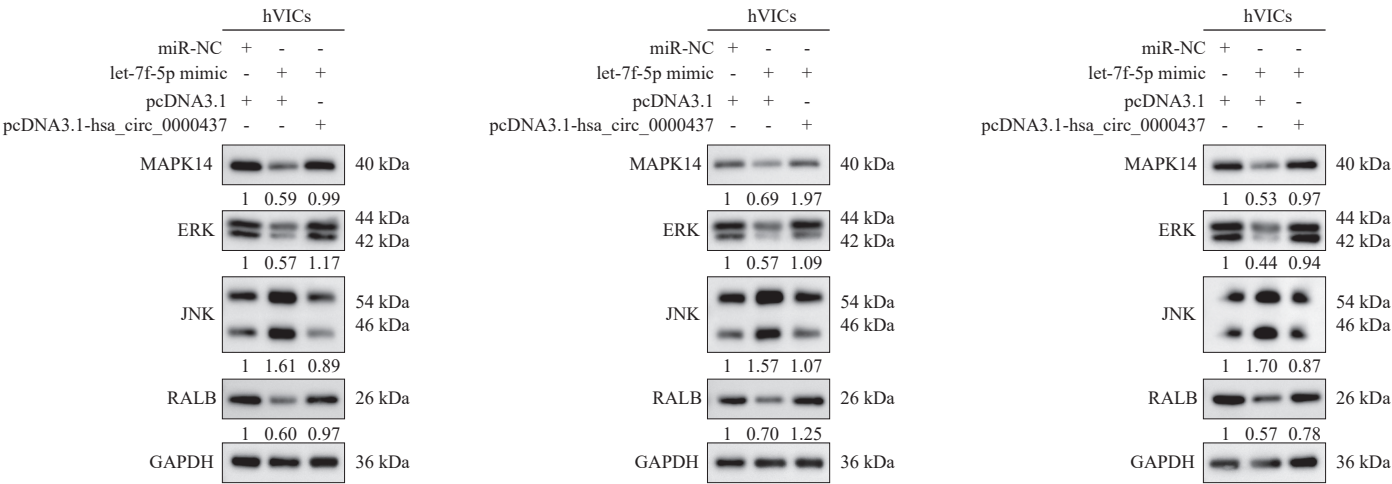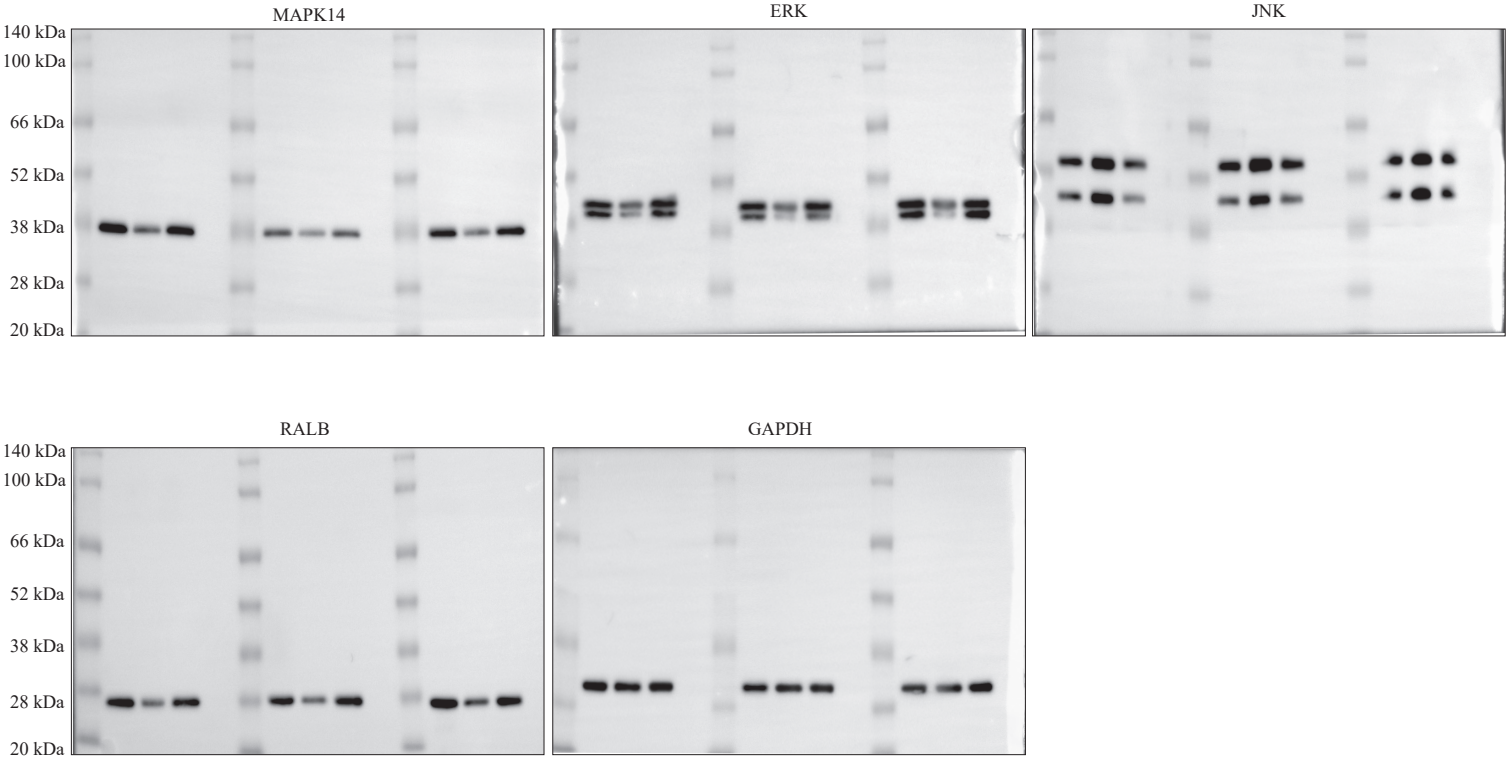

Fig. 7D

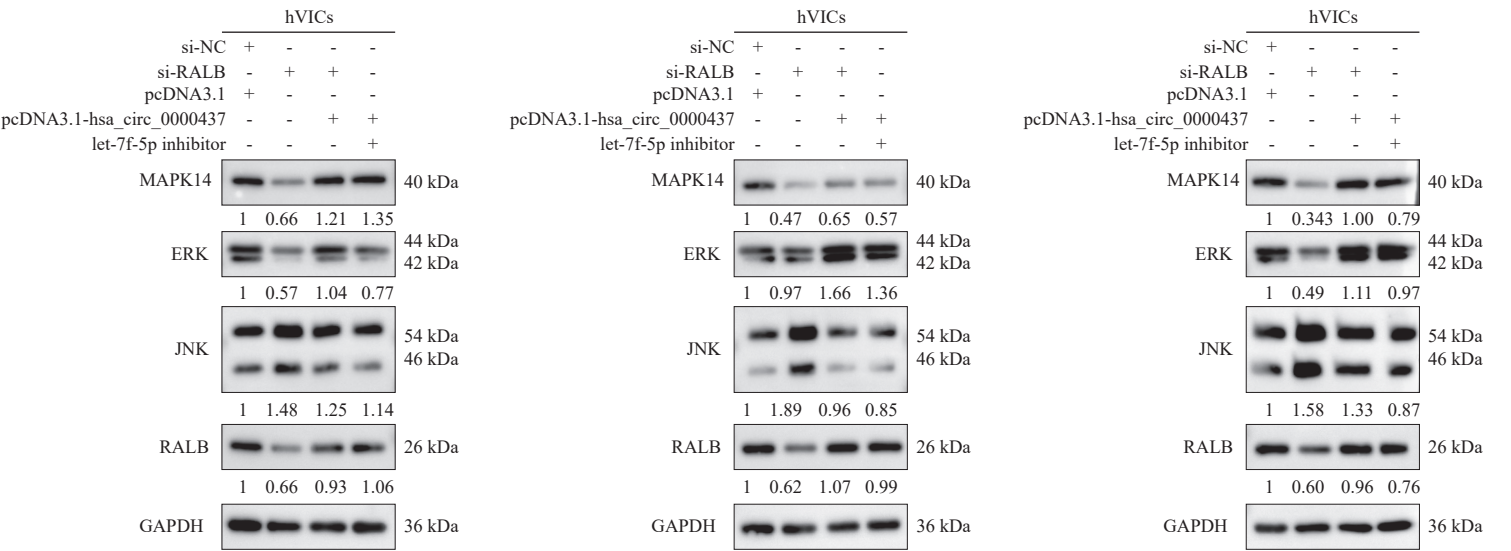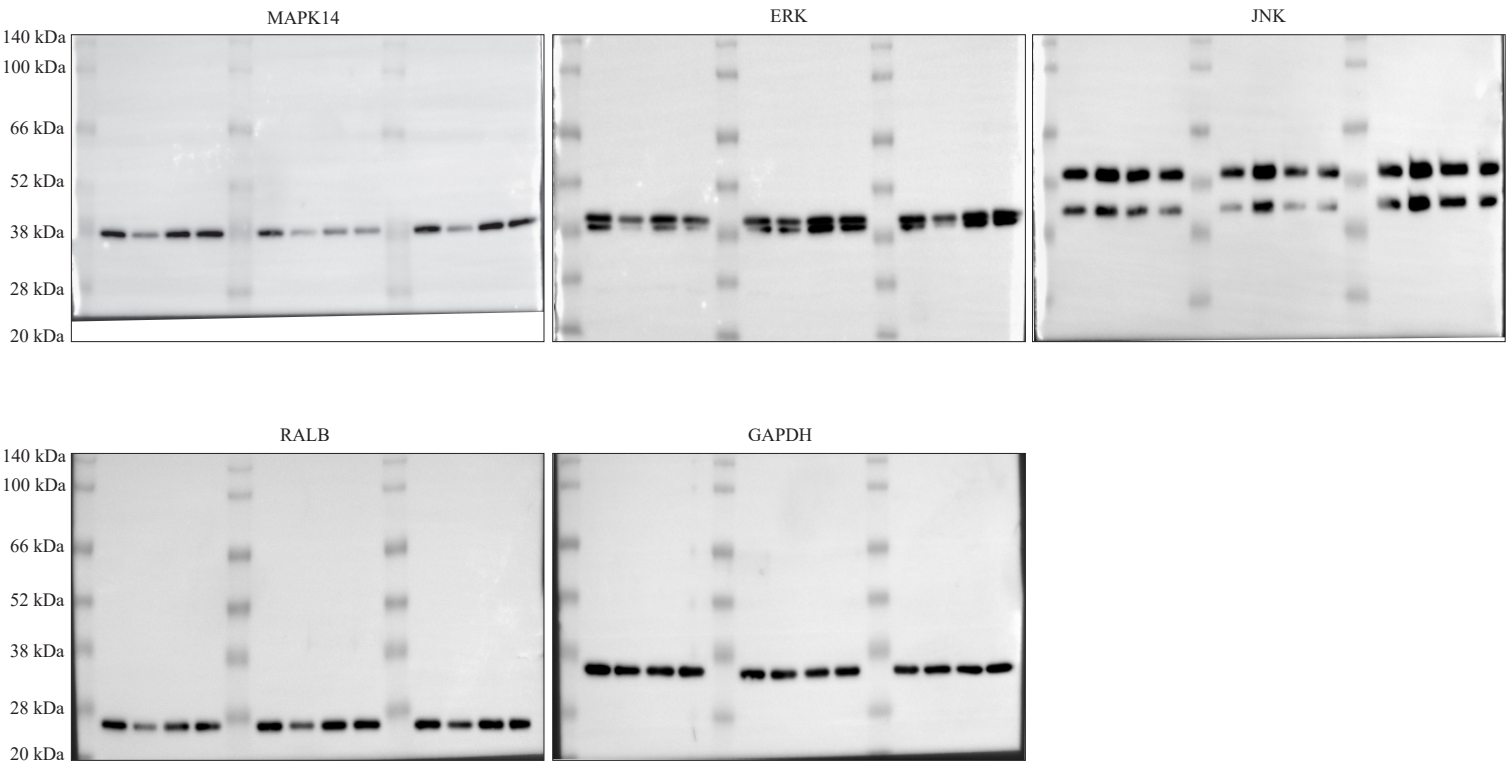

Supplement: Supplementary file 3 — Supplementary file3 (PDF 28017 KB) [file 13577_2025_1331_MOESM3_ESM.pdf]
