# Supplementary material for: Hsa_circ_0000437 promotes the progression of rheumatic valvular heart disease by activating the mitogen-activated protein kinase signaling pathways after sponging let-7f-5p and targeting RAS-like proto-oncogene B
Source: Hum Cell. 2025 Dec 15;39(1):19. doi: 10.1007/s13577-025-01331-7 (PMC12705832; doi:10.1007/s13577-025-01331-7)
Supplement: Supplementary file 4 — Supplementary file4 (DOCX 16 KB) [file 13577_2025_1331_MOESM4_ESM.docx]

Supplementary Table1. Primer and RNA oligonucleotides sequences.

| **Primer** | **Forward primer (5’ to 3’)** | **Reverse primer (5’ to 3’)** |
| --- | --- | --- |
| hsa_circ_0000437(for qPCR)  RALB (for qPCR) | GGGATGGGTTACATGCCCAA  ATGGCTGCAAATAAGCCCAAG | TCCTGCATATTTTTCTGGCAATCTC  TGTCTGCTTTGGTAGGCTCATA |
| GAPDH (for qPCR)  let-7f-5p (for qPCR)  U6 (for qPCR)  let-7f-5p mimic  let-7f-5p inhibitor  si-NC  si-RALB | AAGGTGAAGGTCGGAGTCAA  CGCGCGTGAGGTAGTAGATTGT CAAGGATGACACGCAAATTCG  UGAGGUAGUAGAUUGUAUAGUU  AACUAUACAAUCUACUACCUCA  UUCUCCGAACGUGUCACGUTT  GACCUAAUGAGAGAAAUCAGAACAA | AATGAAGGGGTCATTGATGG  ACGUGACACGUUCGGAGAATT  UUGUUCUGAUUUCUCUCAUUAGGUC |
